# The Key Glutathione S-Transferase Family Genes Involved in the Detoxification of Rice Gramine in Brown Planthopper *Nilaparvata lugens*

**DOI:** 10.3390/insects12121055

**Published:** 2021-11-25

**Authors:** Jun Yang, Xiang-Dong Kong, Keyan Zhu-Salzman, Qing-Ming Qin, Qing-Nian Cai

**Affiliations:** 1 College of Plant Protection, China Agricultural University, Beijing 100193, China; yangjun5123@126.com (J.Y.); xnkongxd@163.com (X.-D.K.); 2 MOA Key Laboratory of Crop Pest Monitoring and Green Control, College of Plant Protection, China Agricultural University, Beijing 100193, China; 3 Department of Entomology, Texas A & M University, College Station, TX 77843, USA; ksalzman@tamu.edu; 4 College of Plant Sciences, Jilin University, Changchun 130062, China; qmqin@jlu.edu.cn; 5 Key Laboratory of Zoonosis Research, Ministry of Education, Jilin University, Changchun 130062, China

**Keywords:** glutathione S-transferases, gramine, *Oryza sativa*, *Nilaparvata lugens*, RNA interference

## Abstract

**Simple Summary:**

Plant defensive compounds are the effective measures for host plants to defend against herbivorous insects, whereas in turn insects evolve an array of detoxifying enzymes to overcome the resistance of these compounds. However, the molecular mechanisms underlying the resistance of plant toxins to herbivores are still unclear. This study uses an integrative approach to investigate the molecular basis of how brown planthopper uses glutathione S-transferases to detoxify gramine, an important defensive toxin in rice. We show here that gramine can induce the activity and increase the transcripts of glutathione S-transferase in brown planthopper. Knockdown of seven glutathione S-transferase family genes of brown planthopper increases its sensitivity to gramine. The glutathione S-transferase activity is regulated by the expression of three key genes (*NlGST1-1*, *NlGSTD2*, and *NlGSTE1*), as silencing of these genes significantly inhibits this enzyme’s activity. The current study identifies a few key glutathione S-transferase genes involved in the detoxification of gramine in brown planthopper. Our findings unravel the molecular mechanisms of the detoxification of plant defensive chemicals by glutathione S-transferases, providing a new potential pest control strategy that uses the method of RNA interference to deplete this gene family to increase the efficiency of host resistance to herbivores.

**Abstract:**

Phytochemical toxins are considered a defense measure for herbivore invasion. To adapt this defensive strategy, herbivores use glutathione S-transferases (GSTs) as an important detoxification enzyme to cope with toxic compounds, but the underlying molecular basis for *GST* genes in this process remains unclear. Here, we investigated the basis of how *GST* genes in brown planthopper (BPH, *Nilaparvata lugens* (Stål)) participated in the detoxification of gramine by RNA interference. For BPH, the LC_25_ and LC_50_ concentrations of gramine were 7.11 and 14.99 μg/mL at 72 h after feeding, respectively. The transcriptions of seven of eight *GST* genes in BPH were induced by a low concentration of gramine, and GST activity was activated. Although interferences of seven genes reduced BPH tolerance to gramine, only the expression of *NlGST1-1*, *NlGSTD2*, and *NlGSTE1* was positively correlated with GST activities, and silencing of these three genes inhibited GST activities in BPH. Our findings reveal that two new key genes, *NlGSTD2* and *NlGSTE1*, play an essential role in the detoxification of gramine such as *NlGST1-1* does in BPH, which not only provides the molecular evidence for the coevolution theory, but also provides new insight into the development of an environmentally friendly strategy for herbivore population management.

## 1. Introduction

Understanding interactions between herbivorous insects and their host plants is challenging when insects colonize. During feeding, herbivorous insects are exposed to an array of plant defensive compounds such as alkaloids and phenolics [1,2,3]. Of them, alkaloids are important nitrogenous compounds in plants, and many alkaloids have been developed as potential pesticides [4,5]. Gramine (indol-3-ylmethyldimethylamine), a simple indole alkaloid, exists widely in wheat *Triticum aestivum*, rice *Oryza sativa*, barley *Hordeum vulgare*, *Arundo donax,* and other gramineous plants [3,6,7,8,9]. Previous studies have reported that this compound is a deterrent and/or toxicant to some herbivorous insects, such as brown planthopper (BPH, *Nilaparvata lugens*), aphids *Rhopalosiphum padi* and *Sitobion avenae*, bettle *Scolytus multistriatus*, and cotton bollworm *Helicoverpa armigera* [3,7,9,10,11,12]. The resistances of gramineous plants to herbivores are highly associated with gramine content in plants. For example, in aphid-resistant wheat varieties and BPH-resistant rice varieties, the contents of gramine are generally higher than that of in the susceptible varieties [3,6,9], leading to the reduction of the survival rate and growth rate of insects after being fed on these resistant varieties. Therefore, herbivores must deal with these defensive compounds in plants properly so as to better adapt to their hosts.

The antagonistic compounds of plants are usually toxic to insects [13], and this toxic effect on insects is closely related to the levels of the secondary metabolites in host plants [9,14]. Although herbivorous insects harbor a large set of these detoxification enzymes, such as glutathione *S*-transferases (GSTs), cytochrome P450 (CYP450s), and carboxylesterases (CarEs), in most cases only a small number of detoxifying enzymes can metabolize specific plant compounds [15]. Of them, insect GSTs belong to one of the important detoxification-related enzymes that convert toxic phytochemicals into nontoxic products, in order to avoid potential damage to insect cellular processes [14,16]. When herbivorous insects are exposed to relatively low concentrations of xenobiotics, induced GST activity in insects rapidly catabolizes these compounds to avoid toxicity accumulation [9,13,17,18,19,20]. Interestingly, not all members of GST family in insects are involved in the detoxification of identical xenobiotic classes. For example, fruit fly *Drosophila melanogaster DmGSTS1-1* and *DmGSTD1* are responsible for the detoxification of mustard oils and 4-hydroxynonenal, respectively [21,22]; BPH *NlGST1-1* is involved in detoxification of gramine and pyrethroid [9,23]. *LsGSTe1* and *LsGSTm* are required for the metabolism of insecticides in *Laodelphax striatellus* [24]. Notably, *GSTs* appear to have substrate specificities since detoxification of some specific xenobiotics largely depends on certain *GSTs* in the GST family [21,25,26,27]. It is thus foreseeable that identifying these detoxification enzyme genes and blocking their functions would reduce the adaptation of these insects to the toxic phytochemical-containing plants.

BPH is a devastating phloem feeding insect that is widely distributed throughout rice production areas in Asia [28]. Feeding exclusively on rice, BPH directly results in losses of rice yield and straw (bioethanol feedstock) by extracting nutrients [29]. BPH-resistant properties of many rice varieties have been quickly overcome due to BPH biotype development [30,31,32]. It is thus necessary to understand the mechanism and factors that are responsible for manifesting the resistance into the selected cultures with desirable characters so that they can be utilized effectively in the breeding program. Perhaps, BPH that can rapidly adapt to their host plants may be closely related to strong detoxification capability in the insect. GSTs in BPH are important detoxification enzymes that are involved in the degradations of toxic rice compounds [9,19]. Previous evidence indicates that *GST* genes in BPH are currently divided into six subfamilies: Delta (*NlGST1-1*, *NlGSTD1,* and *NlGSTD2*), Epsilon (*NlGSTE1*), Sigma (*NlGSTS1* and *NlGSTS2*), Omega (*NlGSTO1*), Theta (*NlGSTT1*), Zeta (*NlGSTZ1*), and a microsomal class [23,27,33]. Of them, eight cytosolic *GST* genes (*NlGST1-1*, *NlGSTD1*, *NlGSTD2*, *NlGSTE1*, *NlGSTO1*, *NlGSTS1*, *NlGSTS2*, and *NlGSTT1*) are highly expressed in midgut and Malpighian tubules, which are the main toxic metabolic organs in BPH [23,27]. Although previous studies have reported that *NlGST1-1* is required for detoxifying pyrethroid and gramine, *NlGSTD1* and *NlGSTE1* are required for detoxifying ferulic acid, and even some *GST* genes are important in imidacloprid resistance [9,19,23,34], while the molecular basis of how BPH GST family is involved in the detoxification of phytochemicals, especially for gramine of a simple indole alkaloid in rice, remains obscure.

In this work, we re-examined the toxicity of gramine to BPH, and investigated the relationship between rice gramine and BPH GST family genes using the method of RNA interference (RNAi). The GST family genes *NlGSTD2* and *NlGSTE1* in BPH play an indispensable role in the detoxification of gramine, the same as *NlGST1-1*. Our research on the coupling of phytochemicals with depletion of BPH detoxification genes provides an efficient management strategy for sap-sucking pests in rice.

## 2. Materials and Methods

### 2.1. Plants and Insects 

Rice BPH (biotype II) nymphs were obtained from paddy fields in Hubei Province, China. The nymphs were reared on five-leaf stage of susceptible *Indica* Taichung Native 1 (TN1) rice seedlings in a climate-controlled chamber at 27 ± 2 °C, 90% RH, and 16 h light/8 h dark (unless otherwise indicated). BPH population was reproduced for at least ten generations on TN1 seedlings before they were used in the subsequent experiments.

### 2.2. Bioassay on Gramine Toxicity to BPH

To understand the toxicity of gramine to BPH in more detail, the lethal concentration 25% (LC_25_) and lethal concentration 50% (LC_50_) of gramine were determined following a previously described method [9,35]. BPH artificial diet was prepared according to the previously described methods [35]. Gramine (purity 99%, CAS Number: 87-52-5, Sigma-Aldrich, St. Louis, MO, USA) was distributed into the liquid artificial diet with concentrations of 0.0 (control), 3.0, 6.0, 12.0, and 24.0 μg/mL. The concentration of gramine used in this experiment was approximately equaled to its content in fresh rice leaves, which ranged from 3 to 140 μg/g wet weight [9,36]. The diet packet holding 100 μL of the liquid diet in double layers of stretched Parafilm M (Pechiney Plastic Packaging Company, Chicago, USA) was placed at one end of a transparent cylindrical glass tube (L × D: 10 cm × 2.5 cm, open at both ends) and per diet packet was fed by fifteen BPH nymphs (second or third instar). The other end of the feeding tube was covered with a piece of gauze to allow air to exchange. Each gramine concentration included 3 feeding tubes with 4 replicates. Diets and feeding sachets were changed daily to avoid diet deterioration and microbial contamination. After feeding for 72 h, dead BPH nymphs in different treatments were recorded for LC_25_ and LC_50_ calculations. The surviving nymphs were collected for the GST activity assay.

To test the impact of nonlethal doses of gramine on each gene expression in BPH GST family, second- or third-instar BPH nymphs were cultured on artificial diets without or with gramine following described methods above. Referring to nonlethal doses of gramine to BPH, 0.0, 2.0, or 3.0 μg/mL gramine was distributed into the diets as control and treatments with 4 replicates. After feeding for 72 h, surviving nymphs were collected for the expression level analysis by quantitative real-time PCR (qRT-PCR).

### 2.3. GST Activity Assay

GST activity was evaluated using a previously described method with minor modification [37]. Ten frozen BPH nymphs in 1 mL of 0.1 M phosphate buffer (pH 6.5) at 4 °C were homogenized, centrifuged, and then the supernatants were collected to analyze the GST activity. The reaction mixture consisted of 0.95 mL of 0.1 M phosphate buffer (pH 6.5), 20 μL of 50 mM 1-chloro-2, 4-dinitrobenzene (CDNB), and 20 μL of 50 mM L-glutathione (reduced). Ten microliters of the supernatant were quickly added into the reaction mixture and monitored the change of the absorbance using a spectrophotometer at 340 nm (SP-756P, Shanghai Metash Instruments Co. Ltd., Shanghai, China). The supernatant was replaced with the same volume of phosphate buffer as the control. GST activity was expressed as μmol·min^−1^·mg protein^−1^ as calculated by using the extinction coefficient for CDNB (9.6 mM^−1^·cm^−1^).

The concentrations of total proteins were determined using a previously described method [38]. The absorbance of the reaction mixture was measured at 595 nm with a spectrophotometer. The standard curve of the protein concentration of BPH samples was established according to the known concentration of bovine serum protein (Sigma-Aldrich, St. Louis, MO, USA).

### 2.4. qRT-PCR

Total RNA of the BPH nymphs was extracted using RNeasy Mini Kit (QIAGEN, Hilden, Germany), and cDNA was prepared by the FastQuant RT Kit (with gDNase; TIANGEN, Beijing, China) following the manufacturer’s instructions. qRT-PCR was performed on an Applied Biosystems (ABI) 7500 Real-Time PCR System (ABI, Foster City, CA, USA) using SYBR *Premix Ex Taq* II (Tli RNaseH Plus; TaKaRa, Shiga, Japan). Due to midgut and Malpighian tubules being the main toxic metabolic organs in BPH, we selected eight cytosolic *GST* genes (*NlGST1-1*, GenBank No. AF448500.1; *NlGSTD1*, GenBank No. JQ917469.1; *NlGSTD2*, GenBank No. JQ917467.1; *NlGSTE1*, GenBank No. JQ917470.1; *NlGSTO1*, GenBank No. JQ917471.1; *NlGSTS1*, GenBank No. JQ917468.1; *NlGSTS2*, GenBank No. JQ917474.1; *NlGSTT1*, GenBank No. JQ917472.1) that were highly expressed in these two tissues as target genes [23,27]. The specific primer sequences of BPH *GST* genes are listed in Table S1. The relative expression levels of target genes were calculated according to the 2^–ΔΔCt^ method [39]. BPH *β-actin* (GenBank No. EU179846) was used as reference gene.

### 2.5. Double-Stranded RNA (dsRNA) Synthesis and Feeding Assays 

The methods of total RNA extraction and cDNA synthesis of the BPH nymphs are described above. Eight aforementioned BPH *GST* genes (*NlGST1-1*, *NlGSTD1*, *NlGSTD2*, *NlGSTE1*, *NlGSTO1*, *NlGSTS1*, *NlGSTS2*, and *NlGSTT1*) were PCR-amplified, ligated into the p*EASY*-T1 vector (TransGen Biotech, Beijing, China), and transformed into *Trans*1-T1 phage resistant chemically competent cell (TransGen Biotech, Beijing, China). After sequencing, the recombinant p*EASY*-*GST* plasmids were served as PCR templates to produce the templates for the synthesis of each *GST* dsRNA with the specific primers containing T7 promoter sequences. The specific primer sequences are listed in Table S1. Each *GST* dsRNA was generated by using the HiScribe T7 in vitro Transcription Kit (NEB, Ipswich, MA, USA) and purified with the RNA Clean Kit (TIANGEN, Beijing, China) following the manufacturer’s instructions. *GFP* (green fluorescent protein, GenBank No. AAX31732.1) dsRNA was synthesized as control.

The dsRNAs of eight *GST* genes were separately dissolved in artificial diets at concentrations of 0.2, 0.4, and 0.8 µg/mL. Among these, *NlGST1-1* dsRNA was used as a positive control and *GFP* dsRNA at 0.4 µg/mL served as a negative control [9]. Feeding tubes containing dsRNAs were assembled, as described in the methods above; each tube was fed with fifteen second- or third-instar BPH nymphs. Each treatment contained 3 sample sizes and 4 replicates. Dead nymphs were recorded for calculating BPH mortality rates. The surviving nymphs were collected to analyze the transcription level of *GST* genes and GST activity, as described above.

### 2.6. Cotreatment of dsRNA and Gramine

First, to determine the nonlethal doses of each *GST* dsRNA to BPH nymphs, each *GST* dsRNA was dissolved into artificial diets at concentrations of 0.05, 0.1, 0.2, and 0.4 µg/mL. *GFP* dsRNA at 0.4 µg/mL was used as control. Fifteen second- or third-instar BPH nymphs were fed in the feeding tubes. The tube containing dsRNAs was performed as described above. The experiment was repeated 3 times. Dead nymphs were recorded for calculating nonlethal doses of each *GST* dsRNA to BPH.

Second, we used the nonlethal doses of each *GST* dsRNA and gramine to evaluate the effect of each gene interference on BPH sensitivity to gramine. The nymph mortalities among *GFP* dsRNA (negative control), dsRNA (positive control), gramine (positive control), and cotreatment of dsRNA and gramine were compared to determine BPH sensitivity to gramine. Ninety BPH nymphs for each construct, divided into 6 feeding tubes (15 nymphs/tube), were fed with diets containing the following nonlethal doses of dsRNA. The same concentration of *GFP* dsRNA as above was used as a control. Three days later, while half of the insects (3 feeding tubes) in each treatment continued to ingest dsRNA-containing diets for additional 3 days, so-called dsRNA control, the other half were provided with gramine-containing diet (3.0 μg/mL) and also fed for 3 more days, denoted as “dsRNA + gramine” (Figure S1). Nymphs that spent the first 3 days on artificial diet only and then transferred to gramine-containing diet for another 3 days functioned as gramine control. In addition, *GFP* dsRNA was considered as negative control (Figure S1). Each treatment contained 3 sample sizes and 5 replicates. Another 3 days later, dead nymphs of all treatments and controls were used to calculate their mortalities.

### 2.7. Statistical Analysis

Statistical analyses were performed using IBM SPSS Statistics version 20.0 (SPSS 20.0) (IBM Inc., Chicago, IL, USA). LC_25_ and LC_50_ of gramine to BPH were calculated by Probit analysis using SPSS 20.0. The least significant difference (LSD) was used in the analysis of variance (*ANOVA*) to indicate significant differences between treatments. All counts of nymph mortality were transformed by *ln* to increase the homogeneity of variances before variance analysis. The correlation of GST activity with the transcriptional level of each gene was assessed by a nonparametric rank correlation test. A probability level of *p* < 0.05 was considered statistically significant.

## 3. Results

### 3.1. LC25 and LC50 of Gramine Toxicity to BPH

**Figure 1:**
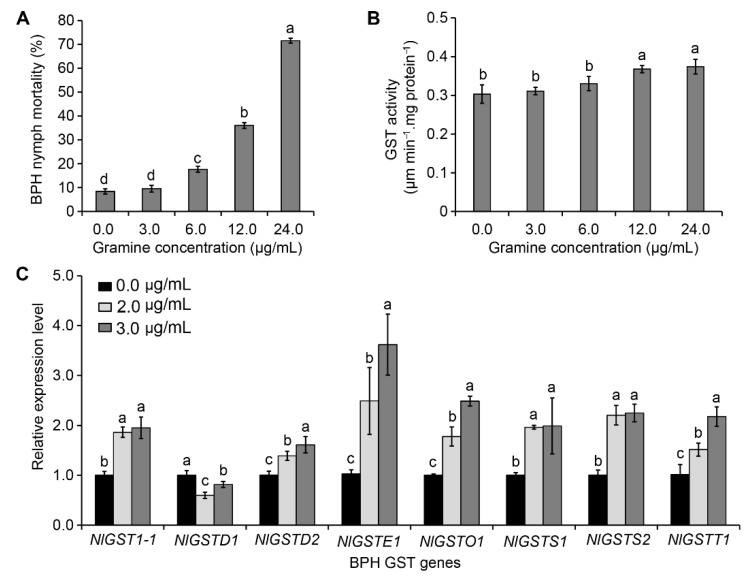
Gramine toxicity to BPH and effect of gramine on BPH nymph *GST* gene expression levels and GST activity. (**A**) Nymph mortality of BPH to gramine. (**B**) GST activity of BPH nymphs fed with different concentrations of gramine. (**C**) The expression level of *GST* gene in BPH nymphs after feeding on diets containing a nonlethal dose of gramine. Data are presented as mean ± standard error of mean (SEM) from 4 replicates. Different letters labeled indicate significant differences at *p* < 0.05.

Bioassay of gramine toxicity to BPH in vitro indicates that increased toxicity to BPH depended on gramine concentrations at 72 h post feeding. The dose-response curves showed that the threshold of gramine observably caused the death of nymphs was 6.0 μg/mL (Figure 1A). Compared with the control, ingestion of 3.0 μg/mL gramine was not sufficient to cause death of BPH. Therefore, this concentration of 3.0 μg/mL gramine was considered as a nonlethal concentration for BPH (Figure 1A). Furthermore, using the Probit scale, we found LC_25_ and LC_50_ of gramine to BPH were 7.11 μg/mL (95% confidence interval: 5.07–9.16 μg/mL) and 14.99 μg/mL (95% confidence interval: 11.57–21.88 μg/mL) at 72 h post feeding, respectively (Table 1). When fed with the diets containing gramine, GST activities can be rapidly induced in BPH, despite the induced GST activity of BPH had no significant differences at the low concentration of gramine (3.0 μg/mL) compared with control (Figure 1B). The activation of GST in BPH by gramine was dose-dependent; the threshold dose for significantly activating BPH was 12.0 μg/mL (Figure 1B).

### 3.2. Effect of Gramine on NlGST Expression in BPH

The nonlethal dosages (≤3.0 μg/mL) of gramine under LC_25_ were used to verify the impact of gramine on each gene expression in the BPH GST family. Dietary gramine (2.0 and 3.0 μg/mL) significantly induced expression levels of seven *GST* genes, *NlGST1-1*, *NlGSTD2*, *NlGSTE1*, *NlGSTO1*, *NlGSTS1*, *NlGSTS2*, and *NlGSTT1*, increasing percentage from 36% to 214% (Figure 1C). Of them, after being fed with 3.0 μg/mL gramine, the expression level of BPH *NlGSTE1* was largely increased by 214%, expression levels of others (*NlGST1-1*, *NlGSTO1*, *NlGSTS1*, *NlGSTS2*, and *NlGSTT1*) also increased by more than 90% (Figure 1C). However, the transcriptional level of *NlGSTD1* was suppressed in the presence of gramine (Figure 1C). The findings indicate that the expression levels of most genes in BPH GST family were significantly raised by gramine, and the sensitivities of these *GST* genes to gramine showed obvious differences.

### 3.3. Correlation between Each GST Interference and GST Activity

**Figure 2:**
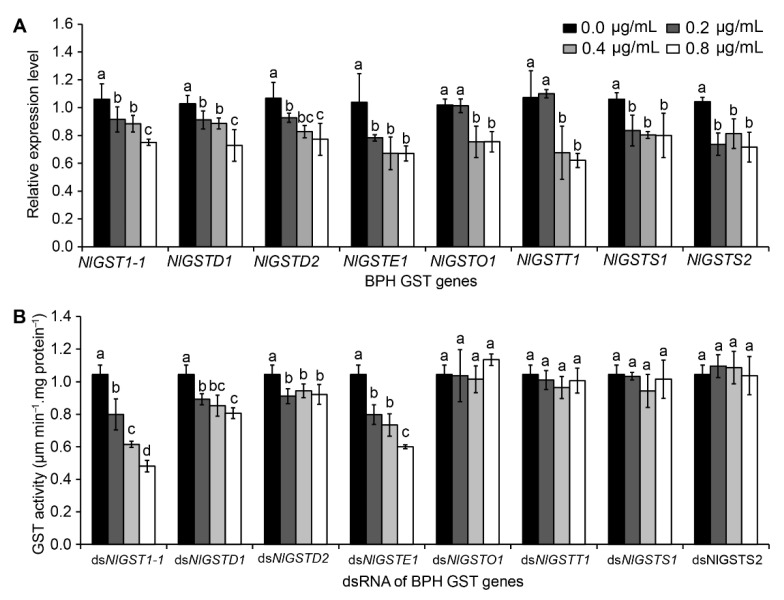
Effect of *GST* dsRNAs on each *GST* gene transcriptional level and GST activity. The second- or third-instar BPH nymphs were fed with artificial diets supplemented with 0 (containing 0.4 μg/mL *GFP* dsRNA), 0.2, 0.4, and 0.8 μg/mL of the indicated dsRNA for 3 days. The transcriptional level of target gene (**A**) and GST activity (**B**) in the nymphs were analyzed. The label “ds + gene” represents the dsRNA of the corresponding *GST* gene. Data represent the means ± SEM from 4 replicates. Different letters labeled indicate significant differences at *p* < 0.05.

**Figure 3:**
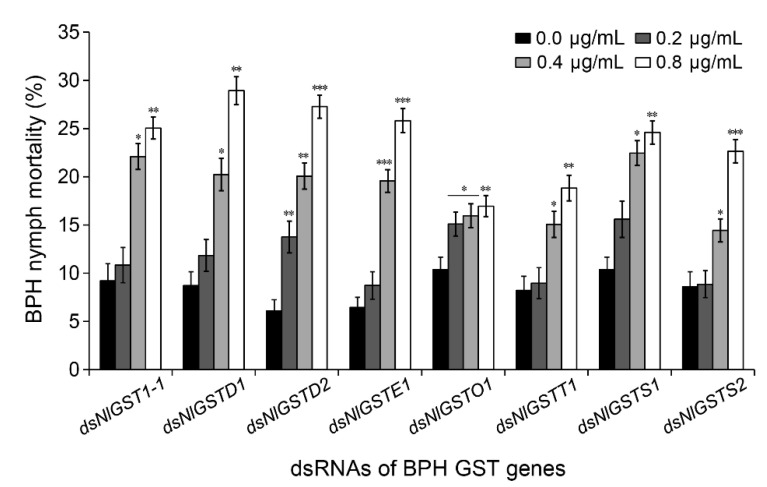
Effect of *GST* dsRNAs on BPH nymph mortality. The second- or third-instar BPH nymphs were fed with artificial diets containing 0.2, 0.4, or 0.8 ng/µL of each *GST* dsRNA or 0.4 ng/µL *GFP* dsRNA (control, without 0.0 ng/µL target gene dsRNA). The nymph mortalities in all the treatments and control were statistically analyzed. The label “ds + gene” represents dsRNA of the corresponding gene. Data represent the means ± SEM from 4 replicates; *, **, *** indicate significance at *p* < 0.05, 0.01, and 0.001, respectively.

To determine whether *GST* transcript levels are correlated with enzymatic activity, we measured the expression level of each *GST* gene and its GST activity after BPH nymphs ingested dsRNAs of individual genes. As expected, the expression level of each gene was significantly reduced by corresponding gene dsRNA (Figure 2A). When fed the diets containing 0.2, 0.4, and 0.8 μg/mL each *GST* dsRNA, the transcript levels of *NlGST1-1*, *NlGSTD1*, *NlGSTD2*, *NlGSTE1*, *NlGSTO1*, *NlGSTT1*, *NlGSTS1*, and *NlGSTS2* in BPHs decreased by 2.60–27.60%, 14.60–21.20%, 5.4–21.40%, 27.80–34.20%, −8.40–27.80%, −9.00–28.25%, −9.75–27.50%, and 16.00–23.20%, respectively (Figure 2A). Although dietary dsRNAs caused transcript reduction of all eight *GST* genes (Figure 2A), only four of them, i.e., *NlGST1-1*, *NlGSTD1*, *NlGSTD2*, and *NlGSTE1*, significantly suppressed the insect GST activity (Figure 2B). Then, we analyzed the correlation between GST activities and transcript levels of these genes, and found that the expression of three genes (*NlGST1-1*, *NlGSTD2*, and *NlGSTE1*) was positively correlated with their GST activities (Table 2). In addition, each *GST* dsRNA with higher concentrations (0.4 μg/mL) significantly increased BPH mortality compared to the *GFP* dsRNA control (Figure 3). 

### 3.4. Suppression of GST Expression Increases BPH Susceptibility to Gramine

**Figure 4:**
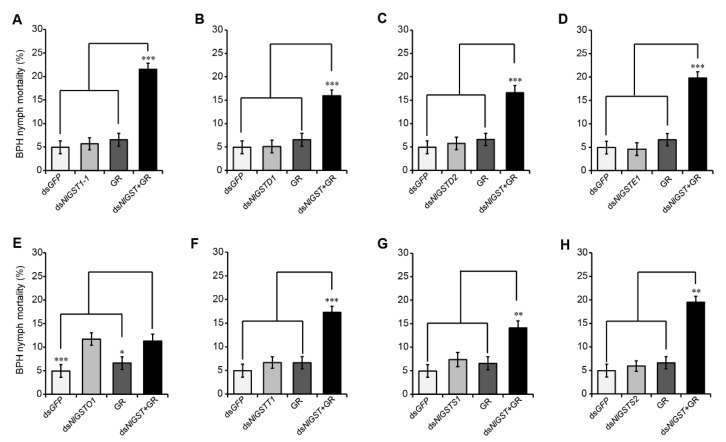
Sensitivity of dsRNA prefed BPH nymphs to gramine-containing diets. BPH nymphs were first fed with the dsRNA-containing diets for 3 days, and then, transferred to new diets with gramine (nonlethal dose) for an additional 3 days, the mortality of the nymphs was used to evaluate the combinative effects of gramine and the dsRNA of *NlGST1-1* (a positive control) (**A**), *NlGSTD1* (**B**), *NlGSTD2* (**C**), *NlGSTE1* (**D**), *NlGSTO1* (**E**), *NlGSTT1* (**F**), *NlGSTS1* (**G**), or *NlGSTS2* (**H**). The label “ds + gene” represents the corresponding dsRNA of each gene; dsGFP: *GFP* dsRNA was used as a control. GR: gramine. Data represent the means ± SEM from 5 replicates. *, **, *** indicate significance at *p* < 0.05, 0.01, and 0.001, respectively.

First, we also defined the nonlethal doses of *GST* dsRNAs to BPH, which means the maximum concentration at which *GST* dsRNAs do not cause significant death for BPH nymphs, compared with the control. There was a difference for the nonlethal doses of each *GST* dsRNAs to BPH nymphs. Of them, the nonlethal doses of *NlGSTD1* and *NlGSTS1* dsRNAs were 0.1 μg/mL; *NlGST1-1*, *NlGSTD2*, *NlGSTS2*, *NlGSTE1* and *NlGSTO1* dsRNAs were 0.2 μg/mL, while only *NlGSTT1* dsRNA was 0.4 μg/mL (Figure S2). Next, to assess the potential effect of silencing BPH *GST* genes on insect detoxification, we recorded mortality of BPH nymphs that fed initially on artificial diets containing the nonlethal doses of each *GST* dsRNAs for 3 days, and then transferred to the nonlethal doses of gramine-containing diet for an additional 3 days (Figure S1). Similar to the positive control (*NlGST1-1*), silencing of six other *GST* genes, namely, *NlGSTD1*, *NlGSTD2*, *NlGSTE1*, *NlGSTS1*, *NlGSTS2*, and *NlGSTT1*, significantly increased the nymph sensitivity to gramine (Figure 4A–D,F–H). The nymph mortality doubled when compared to that of nymphs fed only on gramine (Figure 4A–D,F–H). However, ingestion of *NlGSTO1* dsRNA did not increase the sensitivity to gramine for BPH nymphs (Figure 4E), implying that *NlGSTO1* may be irrelevant to gramine detoxification in BPH. 

## 4. Discussion

Plant secondary metabolites are important defensive compounds [2,3,7,10]. As a result, herbivorous insects possess a wide range of enzymes by which plant-derived toxicants are often metabolized into nontoxic substances [15]. In this study, we investigated each member in BPH GST family association with GST activity, interacting with phytochemical gramine. Besides *NlGST1-1*, BPH GST activity was also closely correlated with *NlGSTD2* and *NlGSTE1* transcript levels; therefore, GST activity in BPH is most likely regulated by a few members in the GST family.

Insects feed on host plants to obtain necessary nutrients, but some toxic compounds from plants can also enter the insect’s body during the feeding process, although their concentrations are low [40,41]. To mitigate the negative impact on their nutritional metabolism, development, and survival, herbivorous insects have evolved counteracting mechanisms such as detoxification, which can be rapidly induced in response to ingestion of toxic compounds, degrading them into nontoxic or less toxic compounds to ensure the normal growth and development of the insects [14,42]. Therefore, blocking detoxification when administrating defensive phytochemicals should be considered as an important strategy for the effective management of key agricultural pests. We have previously demonstrated that rice gramine plays a crucial role in rice resistance to BPH and that the resistance of a rice variety to BPH is highly correlated with its gramine content [9]. We found GST activity of BPH can be induced by the low concentration of gramine in this study, although the previous studies have proved that BPH GST enzyme is an important detoxification enzyme to rice defensive compound—gramine [9,36]. This result is consistent to the previous reports that the GST and carboxylesterase (CarE) activities in *S. avenae* are induced rapidly by ingestion of gramine and ferulic acid, and that of BPH is induced by ferulic acid [9,11,19]; GST activity of *Myzus persicae* can be activated by glucosinolates in host plants [42]. The rapid induction of detoxification enzyme activity by toxic compounds is a common strategy of insect adaptation to plant defense. In addition to GSTs, there are other detoxifying enzymes in insects, such as CYP450s and CarEs, which are also dedicated to the detoxification of toxic compounds including plant defensive compounds and pesticides [19,43,44,45,46,47,48,49]; how each enzyme contributes to the detoxification of the same substance still needs further study.

Elevated GST activities by toxic xenobiotics such as phytochemicals and pesticides in herbivores could result from increased transcriptional levels of key genes encoding these proteins [13,17,18]. The insect GST family is divided into six subfamilies: Delta, Epsilon, Sigma, Omega, Theta, and Zeta [50]. In this study, seven *GST* genes (*NlGST1-1*, *NlGSTD2*, *NlGSTE1*, *NlGSTO1*, *NlGSTS1*, *NlGSTS2*, and *NlGSTT1*) were induced by low content of gramine. Reduction of *NlGST1-1*, *NlGSTD2*, and *NlGSTE1* significantly impaired BPH GST activity, implying that the proteins encoded by these genes may be involved in direct detoxification of gramine. Therefore, suppression of the expression of these gramine-inducible *GST* genes via RNAi approach in BPH increases the sensitivity of the insect to gramine. Interestingly, knockdown of *NlGSTS1*, *NlGSTS2*, or *NlGSTT1* enhanced BPH sensitivity to gramine, but had no effect on the GST activity. Two reasons may support this result: (1) these particular *GSTs* may be having activity with other substrates other than the substrate used in this study, because different substrates can cause great differences in the determination of GST activity [42,51]; (2) these genes may be associated with other physiological pathways in BPH. For example, *AccGSTS1* in *Apis cerana cerana*, *DmGSTS1-1* in *D. melanogaster*, and *TcGSTS6* in *Tribolium castaneum*, are involved in the defense of cellular antioxidants [22,52,53]. In *Anopheles cracens*, *AcGSTT1-1* may be a peroxidase that acts as a binding protein for organophosphates to detoxify the insecticides [54]. Of course, we cannot limit our understanding of these GST subfamilies, because it has been reported that the Sigma subfamily of GST is important for insecticide resistance [24,34]. Consistent with the previous findings [27,54], the involvement of *NlGSTO1* in BPH metabolism and detoxification of xenobiotics was limited. This is because *NlGSTO1* may participate in antioxidant resistance in BPH [55]. Therefore, our results apparently suggest that not all *GST* genes are involved in the detoxification of gramine in BPH, although GST activity in herbivores is closely related to its detoxification capability against xenobiotics [9,18,20].

With the development of RNAi technology, this method has become one of the effective tools for studying the function of insect genes. However, no matter by feeding or injection, during the RNAi reaction, the dsRNA entered in the insect body is processed to RNA segments 21–23 nucleotides in length to produce an RNAi effect [56,57]. Hence, some sequence’s contiguous match that occurs among different insect species is more common in sibling species and less common in distantly related species [36,58,59]. In the GST family, Delta and Epsilon subfamilies are only found in insects [50]; surprisingly, the *GST* genes involved in the detoxification of gramine belonged to these two GST subfamilies in BPH. The white-backed planthopper *Sogatella furcifera* is a sibling species with BPH; the two insects are both important pests of rice, which occur simultaneously in rice, causing serious economic losses. Compared with the sequence similarities of Delta and Epsilon subfamilies of GSTs in BPH and their orthologous genes in *S. furcifera*, we found they share 89.40% nucleotide sequence similarity between *NlGST1-1* and *SfGSTD2*, 89.55% similarity between *NlGSTD2* and *SfGSTD2*, and 81.28% similarity between *NlGSTE1* and *SfGSTE1* (Figure S3). The risk assessment of transgenic rice expressing BPH *NlGST1-1* dsRNA found that forty-six 21-nt (nucleotide) continuous sequence matches in *SfGSTD2* [36]. Thus, we predict that comparable to BPH, the white-backed planthopper *SfGSTD2*, and even the homologous genes that are highly similar to these two species in other insects, could also involve in detoxicating the phytochemicals to be suitable for host plants. If this speculation is reasonable and feasible, the use of orthologous gene dsRNA in insects at least can be a potential tool for controlling the sibling species of BPH, *S. furcifera*. Certainly, future studies evaluating the effects of RNAi, especially plant-mediated RNAi on nontarget organisms, are necessary, and this evaluation will give us a holistic view of the impacts of RNAi on these unintended targets [60,61]. Although the current research in this area is very limited, we attempt to provide a new idea for the control of some sibling species of insects. As for the control effect of using orthologous genes in related species, it needs to be further tackled.

## 5. Conclusions

In summary, we revealed the molecular basis of the BPH GST family genes to the detoxification of gramine, one important rice defensive compound, that *NlGSTD2* and *NlGSTE1* play an indispensable role in the detoxification of this compound in rice. This research suggests that a few key members of GST family are associated with the tolerance of rice defensive compounds in BPH, providing a new approach for high-efficiency management of the sap-sucking insect population. 

## Figures and Tables

**Table 1:** LC_25_ and LC_50_ of gramine against BPH.

*n*	LC_25_ (μg/mL) ^a^	LC_50_ (μg/mL) ^a^	Slope ± SE ^b^	(χ^2^) ^c^	*p*
186	7.11 (5.07–9.16)	14.99 (11.57–21.88)	2.084 ± 0.37	2.71	0.987

^a^ lethal concentration causing 25% and 50% mortality after 3 d with 95% confidence limits; ^b^ slope ± standard error of the concentration–mortality regression line; ^c^ chi square.

**Table 2:** Correlation of *GST* gene transcription level with GST activity in BPH.

*GST* Transcript-GST Activity	*n*	Equation	r	*p*	Significance
*NlGST1-1*-GSTs	4	y = 1.6649x − 0.7865	0.9719	0.0141	*
*NlGSTD1*-GSTs	4	y = 0.3453x + 0.5347	0.7612	0.1194	ns
*NlGSTD2*-GSTs	4	y = 1.1624x − 0.1754	0.9839	0.0080	**
*NlGSTE1*-GSTs	4	y = 0.6451x + 0.2213	0.9541	0.0230	*
*NlGSTO1*-GSTs	4	y = 0.2159x + 0.9205	0.5605	0.2197	ns
*NlGSTS1*-GSTs	4	y = 0.1235x + 0.9928	0.4532	0.2734	ns
*NlGSTS2*-GSTs	4	y = 0.2489x + 0.8809	0.8772	0.0614	ns
*NlGSTT1*-GSTs	4	y = 0.3803x + 0.7793	0.8408	0.0796	ns

*, **: significance at *p* < 0.05 and 0.01, respectively; ns: *p* > 0.05. A bivariate Spearman’s nonparametric rank correlation test was used in the correlation analysis.

## Data Availability

All data discussed in the current work are provided in the article and online Supplementary Materials.

## Supplementary Materials

The following are available online https://www.mdpi.com/article/10.3390/insects12121055/s1, Figure S1. The process diagram of the effects of silencing *NlGSTs* on BPH sensitivity to gramine. The process diagram of the effects of silencing *NlGSTs* on BPH sensitivity to gramine. The second- or third-instar BPH nymphs from rice were introduced into feeding tubes (15 nymphs/tube). Forty-five nymphs distributed into three feeding tubes, were fed with artificial diets (AD) with *GST* dsRNA (AD + dsRNA), *GFP* dsRNA (AD + *GFP* dsRNA), or no dsRNA (AD) for 3 days, and then separately transferred to the new diets with gramine (AD + gramine) and corresponding dsRNA (AD + dsRNA or AD + *GFP* dsRNA) to continue to feed for additional 3 days as the treatments of “dsRNA + gramine”, “dsRNA positive control”, “gramine control”, and “*GFP* dsRNA negative control”, respectively. All treatments and controls were replicated 5 times. Final nymph mortality was used for variance analysis to compare significant difference between treatment and control. Figure S2. The mortality of BPH nymphs to *GST* dsRNAs. Ten second- or third-instar BPH nymphs were fed on artificial diets containing *NlGST1-1* (A), *NlGSTD1* (B), *NlGSTD2* (C), *NlGSTE1* (D), *NlGSTO1* (E), *NlGSTS1* (F), *NlGSTS2* (G), and *NlGSTT1* (H) dsRNA, respectively, for three days. Concentrations of 0.05, 0.1, 0.2, and 0.4 µg/mL for each *GST* dsRNA were used in these experiments; 0.4 µg/mL *GFP* dsRNA was used as control. The mortality of BPH nymphs was recorded and analyzed. Compared with the control, the maximum concentration at which *GST* dsRNA does not cause significant death for BPH nymphs was considered as a nonlethal dose for BPH *GST* dsRNA. Data represent the means ± SD from three replicates. Different letters labeled indicate significant differences at *p* < 0.05. Figure S3. Alignment of Delta and Epsilon subfamilies of GSTs in BPH and their orthologous genes in *Sogatella furcifera*. *NlGST1-1*, *NlGSTD2*, and *SfGSTD2* belong to Delta subfamilies of GSTs, and *NlGSTE1* and *SfGSTE1* are the members of Epsilon subfamilies of GSTs in insects. The homology analysis between *NlGST1-1* and *SfGSTD2* (A), *NlGSTD2* and *SfGSTD2* (B), and *NlGSTE1* and *SfGSTE1* (C) were performed by multiple sequence alignment using the DNAMAN software. Table S1. Primers used in this study.
