# Supplementary material for: The Key Glutathione S-Transferase Family Genes Involved in the Detoxification of Rice Gramine in Brown Planthopper Nilaparvata lugens
Source: Insects. 2021 Nov 25;12(12):1055. doi: 10.3390/insects12121055 (PMC8704333; doi:10.3390/insects12121055)
Supplement: Supplementary file 1 [file insects-12-01055-s001.zip › insects-1404152-supplymentary materials.pdf]

Supplementary materials for

The Key Glutathione S-Transferase Family Genes Involved in the Detoxification of Rice Gramine in Brown Planthopper *Nilaparvata lugens*

Contents:

Figure S1. The process diagram of the effects of silencing *NlGSTs* on BPH sensitivity to gramine.

Figure S2. The mortality of BPH nymphs to *GST* dsRNAs.

Figure S3. Alignment of Delta and Epsilon subfamilies of *GSTs* in BPH and their orthologous genes in *Sogatella furcifera*.

Table S1. Primers used in this study.

Figure S1. The process diagram of the effects of silencing *NlGSTs* on BPH sensitivity to gramine.

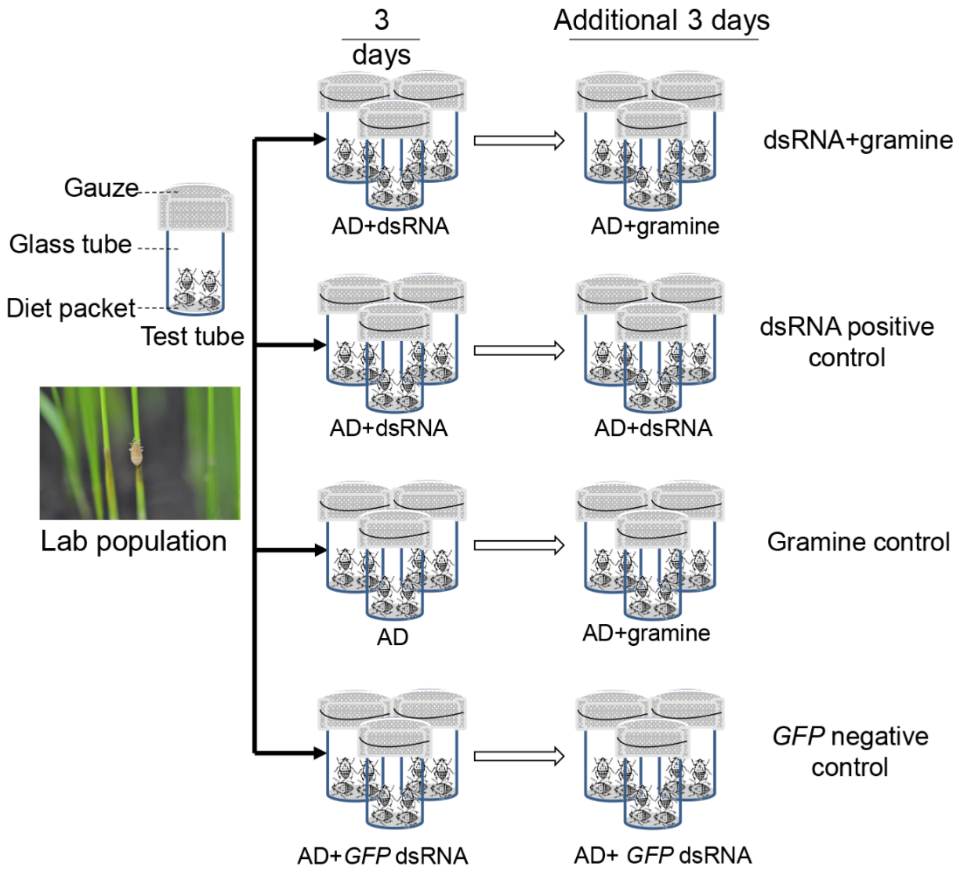

Figure S2. Non-lethal dose of *GST* dsRNAs to BPH nymphs.

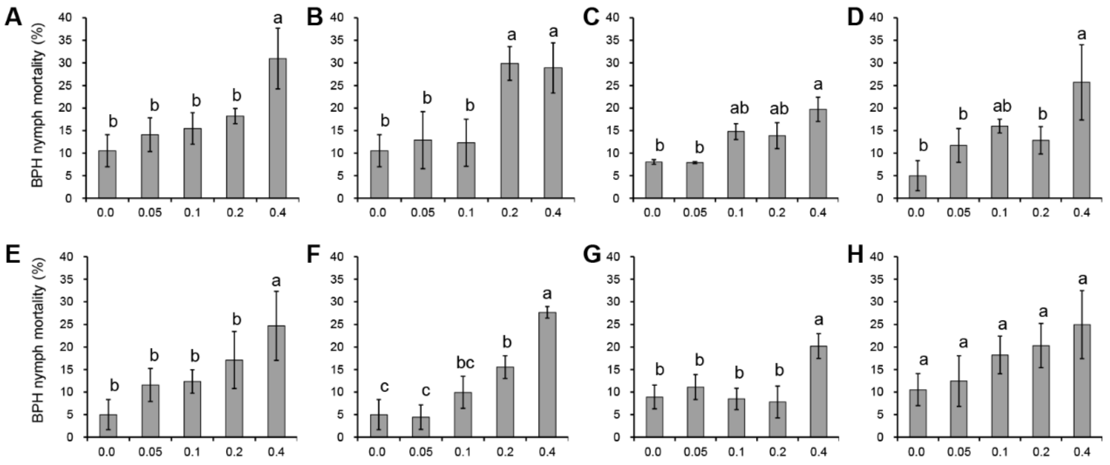

Figure S3. Alignment of Delta and Epsilon subfamilies of GSTs in BPH and their orthologous genes in *Sogatella furcifera*.

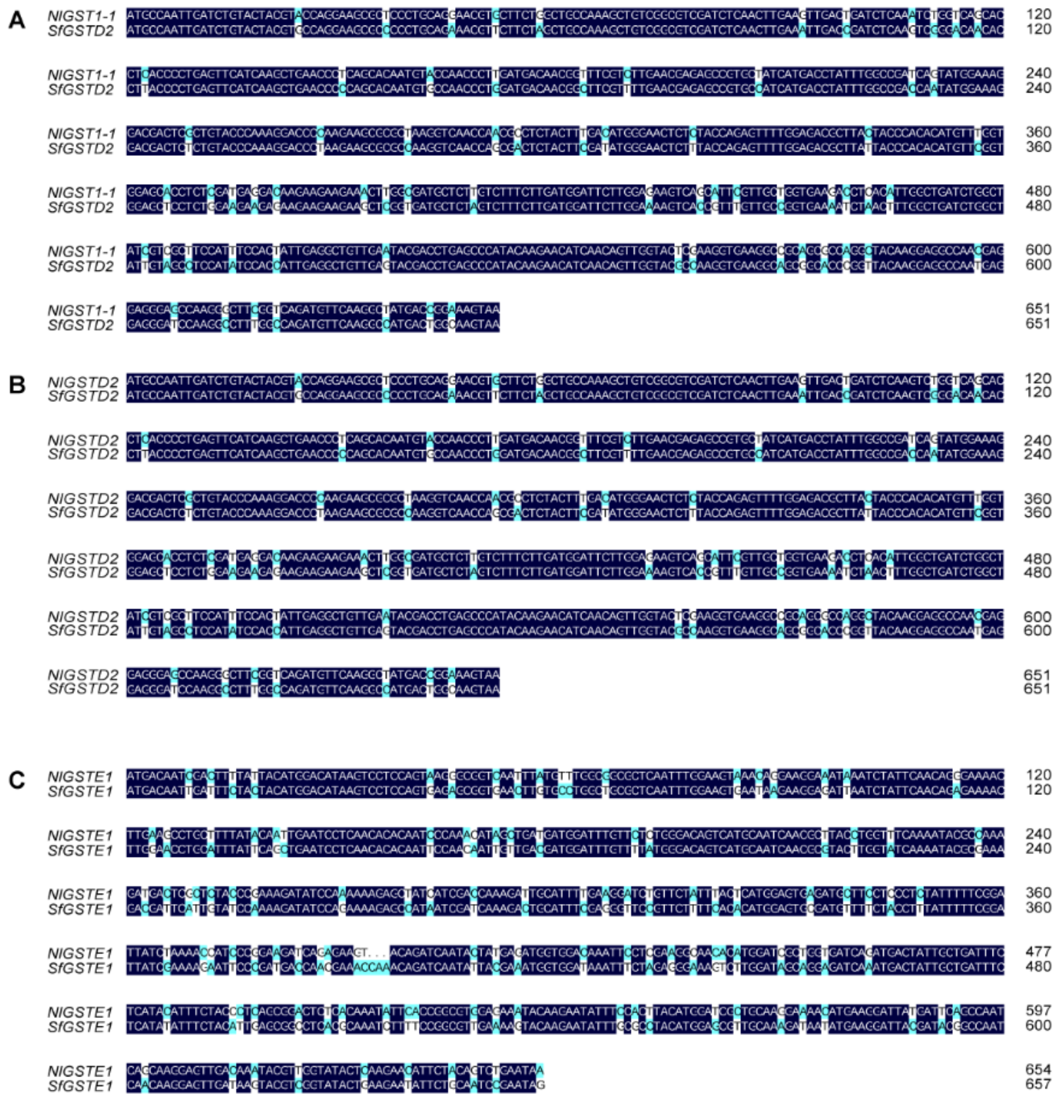

Table S1. Primers used in this study.

Purposes	Primer Names	Sequences (5'-3')
	**qRT-PCR**	
1	qNlGST1-1-F	CTAAGGTCAACCAACGCCTCTACT
	qNlGST1-1-R	GATGTTCTTGTATGGGCTCAGGTC
2[a]	qNlGSTD1-F	CCGAGGAACCCACAACAGAA
	qNlGSTD1-R	CCAAGCCTCACAACTGGGAA
3	qNlGSTD2-F	CTGGCTATCGTCGCTTCCAT
	qNlGSTD2-R	CCTCGTTGGCCTCCTTGTAG
4[a]	qNlGSTE1-F	GGAGTGAGATGCTTCCTCCC
	qNlGSTE1-R	GCGATCCATGTGTTGCCTTC
5	qNlGSTS1-F	GCTGATCTCGCTGGAAAGGA
	qNlGSTS1-R	TTCTTGGTGGCTTCATCGCT
6	qNlGSTS2-F	CCTTACGGCCAAGTTCCTGT
	qNlGSTS2-R	CCCACTCATTACTCCCAGCC
7	qNlGSTT1-F	TGCGATATCTGTGCCGAGAG
	qNlGSTT1-R	TGAGCTCGATGTGTTGCCAT
8	qNlGSTO1-F	TAGTATGCGTTTCTGCCCTTATGC
	qNlGSTO1-R	TTGCTGGTGTACCATTCTGGCTTC
9[a]	qNl-β-actin-F	TGGACTTCGAGCAGGAAATGG
	qNl-β-actin-R	ACGTCGCACTTCATGATCGAG
	**dsRNA**	
10	NlGST1-1-F	ATGCCAATTGATCTGTACTACGTACC
	NlGST1-1-R	TTACTTTCCGGTCATAGCCTTGAAC
11[a]	NlGSTD1-F	ATGGCAGCAGTGACTTTATATC
	NlGSTD1-R	TATCTGCCCCGGATCAACC
12	NlGSTD2-F	ATGCCAATTGATCTGTACTACGTACC
	NlGSTD2-R	CTTTCCGGTCATAGCCTTGAACATC
13[a]	NlGSTE1-F	ATGACAATCGACTTTTATTAC

	NlGSTE1-R	TTCAGACTGTAGAATGTTCTTGAG
14	NlGSTS1-F	ATGGATGATGTCTTACCTGGAT
	NlGSTS1-R	TATACCAGGGAGTCTTTTCAAC
15	NlGSTS2-F	ATGCCTACATATAAGTTGACCTACTAC
	NlGSTS2-R	CTTCTTGGGTACAGGCCGCTTCTCA
16	NlGSTT1-F	ATGAGTGGCCGTCAATCAGCAGTTAC
	NlGSTT1-R	CAATTTTGACACAACCTGAGAAGATCTC
17	NlGSTO1-F	ATGGCTGCGATTGAGCATCTAAC
	NlGSTO1-R	CTAAGCAATGATAACATAGTCGGGAG
18	GFP-F	CTGGAGTTGTCCCAATTCTTGTT
	GFP-R	ATGGTCTGCTAGTTGAACGCTTC
19	T7NlGST1-1-F	TAATACGACTCACTATAGGGATGCCAATTGATCTGTACTACGTACC
	T7NlGST1-1-R	TAATACGACTCACTATAGGGTTACTTTCCGGTCATAGCCTTGAAC
20[a]	T7NlGSTD1-F	TAATACGACTCACTATAGGGATGGCAGCAGTGACTTTATATC
	T7NlGSTD1-R	TAATACGACTCACTATAGGGTATCTGCCCCGGATCAACC
21	T7NlGSTD2-F	TAATACGACTCACTATAGGGATGCCAATTGATCTGTACTACGTACC
	T7NlGSTD2-R	TAATACGACTCACTATAGGGCTTTCCGGTCATAGCCTTGAACATC
22[a]	T7NlGSTE1-F	TAATACGACTCACTATAGGGATGACAATCGACTTTTATTAC
	T7NlGSTE1-R	TAATACGACTCACTATAGGGTTCAGACTGTAGAATGTTCTTGAG
23	T7NlGSTS1-F	TAATACGACTCACTATAGGGATGGATGATGTCTTACCTGGAT
	T7NlGSTS1-R	TAATACGACTCACTATAGGGTATACCAGGGAGTCTTTTCAAC
24	T7NlGSTS2-F	TAATACGACTCACTATAGGGATGCCTACATATAAGTTGACCTACTAC
	T7NlGSTS2-R	TAATACGACTCACTATAGGGCTTCTTGGGTACAGGCCGCTTCTCA
25	T7NlGSTT1-F	TAATACGACTCACTATAGGGATGAGTGGCCGTCAATCAGCAGTTAC
	T7NlGSTT1-R	TAATACGACTCACTATAGGGCAATTTTGACACAACCTGAGAAGATCTC
26	T7NlGSTO1-F	TAATACGACTCACTATAGGGATGGCTGCGATTGAGCATCTAAC
	T7NlGSTO1-R	TAATACGACTCACTATAGGGCTAAGCAATGATAACATAGTCGGGAG
27	T7GFP-F	TAATACGACTCACTATAGGGCTGGAGTTGTCCCAATTCTTGTT
	T7GFP-R	TAATACGACTCACTATAGGGATGGTCTGCTAGTTGAACGCTTC

[a] Reference

Yang, J.; Sun, X.-Q.; Yan, S.-Y.; Pan, W.-J.; Zhang, M.-X.; Cai, Q.-N. Interaction of ferulic acid with glutathione S-transferase and carboxylesterase genes in the brown planthopper, *Nilaparvata lugens*. *J. Chem. Ecol.* **2017**, *43*, 693-702. https://doi.org/10.1007/s10886-017-0859-3.
